# Inferior Vena Cava Masson’s Tumor Extending Into the Right Atrium and Ventricle: A Case Report

**DOI:** 10.7759/cureus.113790

**Published:** 2026-08-01

**Authors:** Gagan Aulakh, Arshdeep Singh

**Affiliations:** 1 Nephrology, University of Massachusetts Chan Medical Center, Worcester, USA; 2 Internal Medicine, Government Medical College Amritsar, Amritsar, IND

**Keywords:** angiosarcoma, cardiac mass, intravascular papillary endothelial hyperplasia (ipeh), ivc mass, masson’s tumor

## Abstract

Intravascular papillary endothelial hyperplasia (IPEH) is an uncommon vascular lesion, typically found on the skin of the head, neck, and extremities. Angiosarcoma remains the most important differential diagnosis and should be excluded based on histopathologic features. The preferred treatment for IPEH is complete surgical excision, as incomplete resection may lead to recurrence. We report the case of a 46-year-old female who was evaluated for chest pain and syncope, revealing a right-sided cardiac mass. There was uncertainty regarding the origin and full extent of the cardiac mass despite transthoracic and transesophageal echocardiography. Transesophageal echocardiography localized the lesion to the inferior vena cava (IVC), while open-heart surgery confirmed its full 14 cm extent and IVC origin. The mass was completely excised, and histopathology was consistent with IPEH (Masson’s tumor). This case presents a rare vascular tumor at an unusual location, which initially posed a diagnostic challenge. However, through a collaborative approach with a multidisciplinary team, we were able to establish the correct diagnosis and successfully prevent recurrence.

## Introduction

Intravascular papillary endothelial hyperplasia (IPEH), also known as Masson’s tumor, is a benign growth typically located on the skin of the head, neck, and extremities. IPEH is a rare entity, accounting for approximately 2% of vascular tumors. However, intracardiac involvement is exceptionally rare, with limited literature available on the subject. Overall, 30% of cases are associated with trauma and vascular malformations, hemangioma, or stasis. However, the cause remains unknown in about 70% of instances [1,2]. It exhibits a higher occurrence in women, with a ratio of 1.3:1. Histologically, IPEH displays endothelial papillary structures organized into a thrombus. Its main point of differentiation from angiosarcoma, which is malignant in nature, lies in the absence of necrosis, cellular atypia, and mitotic figures and the presence of positive CD31, CD34, factor VIII-related antigen, and smooth muscle actin. The use of three-dimensional echocardiography and contrast aids in the differentiation, as contrast is taken up by malignant cells, leading to the absence of uptake by IPEH [1,2]. It is important to distinguish IPEH from angiosarcoma because the treatment differs significantly. While complete surgical excision is the treatment of choice for IPEH, angiosarcoma is a malignant tumor that often requires chemotherapy in addition to surgical resection [2]. IPEH has a higher risk of recurrence if it is not completely resected [1,2].

## Case presentation

**Figure 1:**
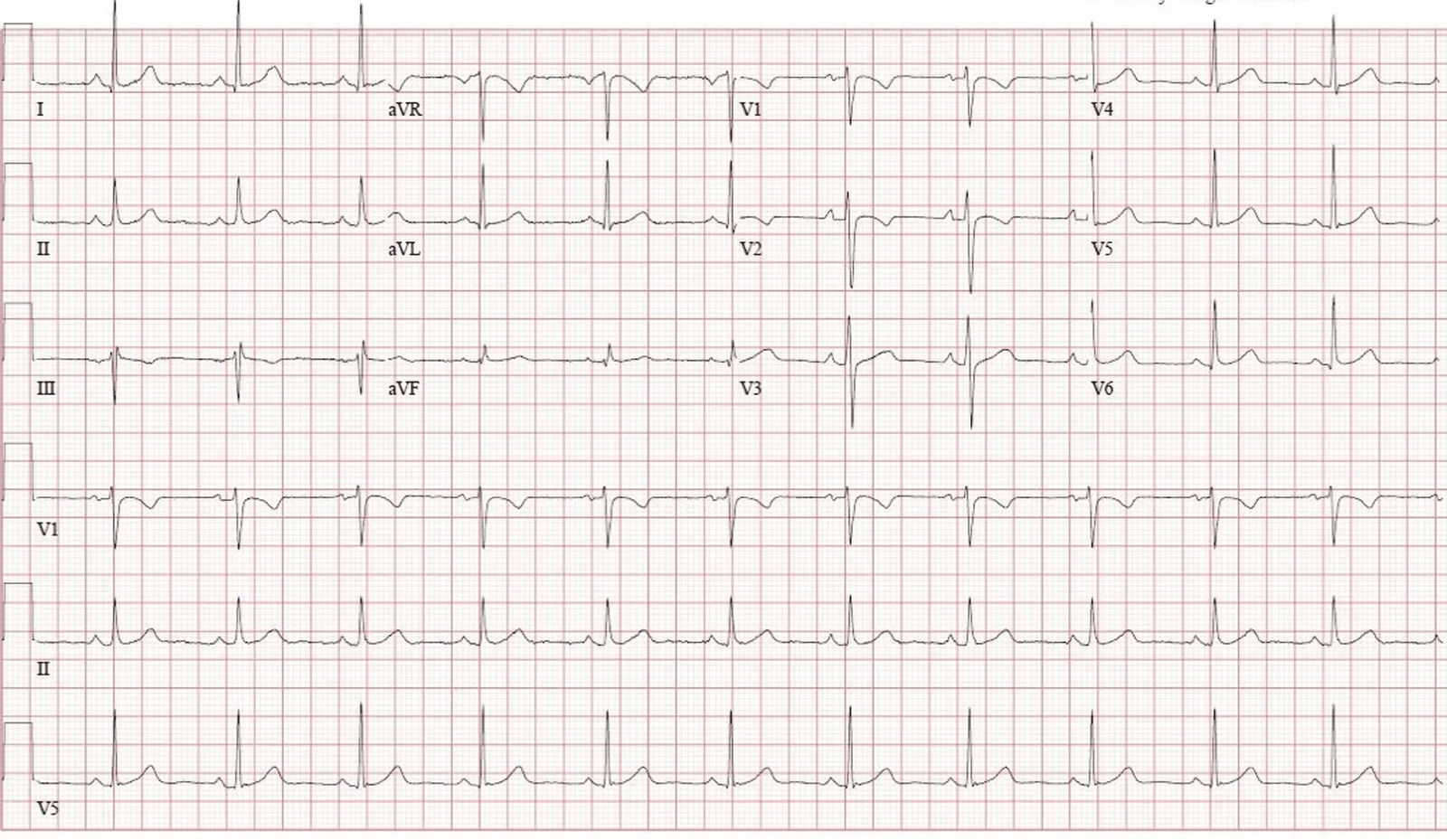
Electrocardiogram at presentation revealing normal sinus rhythm with unremarkable findings.

A 46-year-old female with no significant past medical history was admitted for evaluation due to chest pain and syncopal events. She had experienced two exertional syncopal events in the past month, accompanied by diaphoresis and preceded by dizziness. She had been experiencing central intermittent exertional chest pain over the same period. She denied any dyspnea, palpitations, postictal confusion, or incontinence. There was no family history of sudden cardiac death or premature coronary artery disease. The examination revealed no significant findings, including any murmurs. Vital signs showed a blood pressure of 119/76 mmHg, heart rate of 80 beats/minute, respiratory rate of 20 breaths/minute, and SpO_2_ of 95% in room air. Electrocardiogram showed normal sinus rhythm without any ischemic or pericarditis changes (Figure 1).

Our list of differential diagnoses included pulmonary embolism, coronary artery disease, paroxysmal arrhythmias, and bicuspid aortic valve or valvular obstruction. The CT pulmonary angiography revealed negative results for pulmonary embolism. The transthoracic echocardiogram indicated the presence of a large multilobed right atrial mass, likely originating from the interatrial septum. This mass appeared protruding into the right ventricle through the tricuspid valve during diastole. Given the mobility of the mass and its apparent extension through the tricuspid valve, intermittent obstruction of tricuspid inflow was considered a possible mechanism contributing to her syncopal episodes. However, the exact etiology remained uncertain. These findings expanded our list of potential diagnoses, including mural thrombus, myxoma, angiosarcoma, or fibroma.

Subsequently, we commenced heparin drip for stroke prophylaxis. For better visualisation, transesophageal echocardiography (TEE) was planned, and the cardiothoracic surgery team was consulted. The TEE revealed a completely different image, indicating a multilobulated mass measuring 4 cm × 1.1 cm that originated from the inferior vena cava (IVC) extending to the right atrium through the right ventricle with no uptake of contrast. Out of the total length of 4 cm, 2.7 cm was located within the IVC, exhibiting a serpentine extension suggestive of a stalk (Video 1).

**Video 1:** Transesophageal echocardiography demonstrating a highly mobile multilobulated right-sided intracardiac mass extending between the right atrium and right ventricle through the tricuspid valve. Color Doppler imaging demonstrates the absence of internal vascular flow within the lesion.

**Figure 2:**
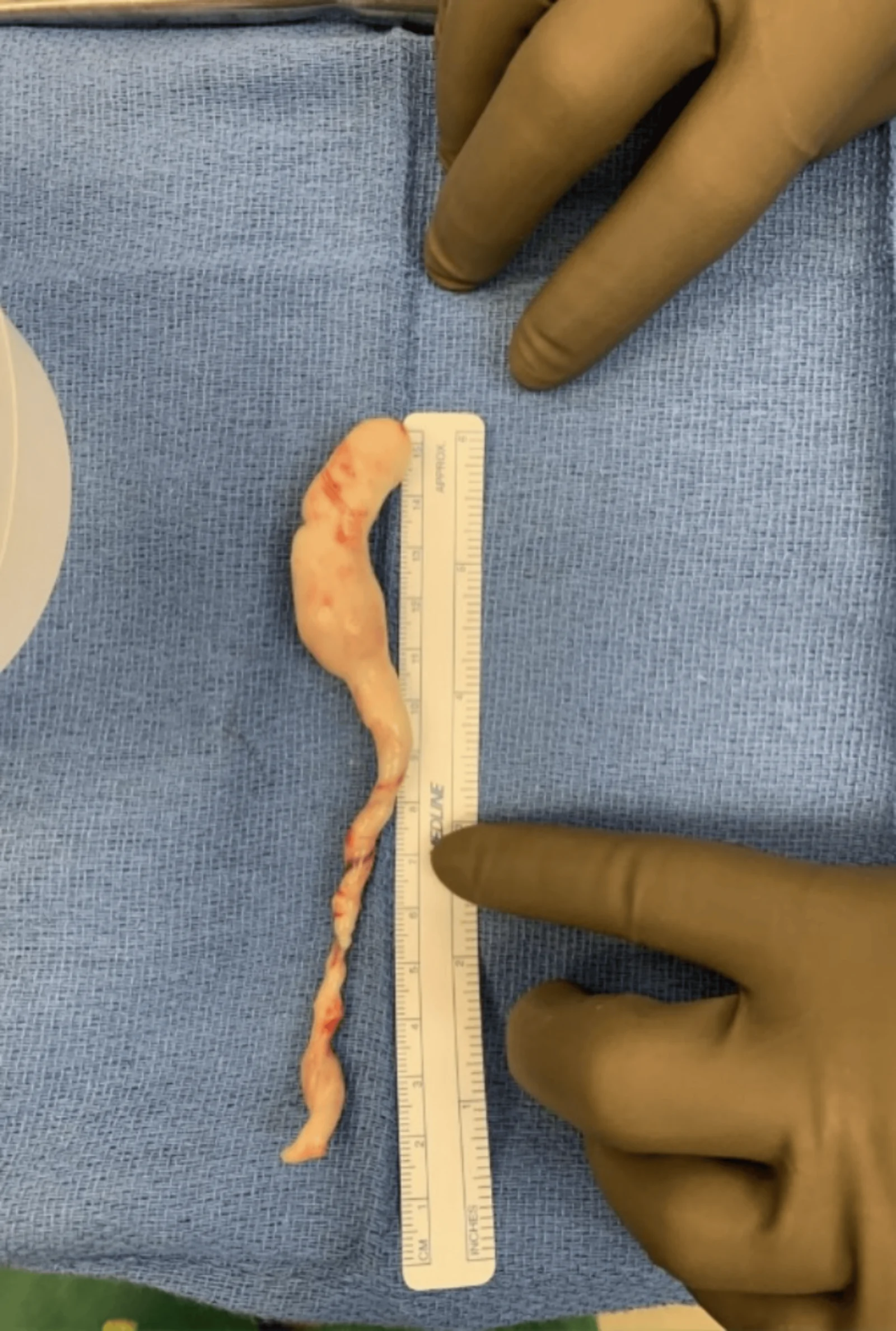
Gross specimen of the excised inferior vena cava (IVC) mass. Surgical specimen demonstrating a pedunculated intravascular mass attached by a stalk and removed en bloc from the IVC.

The cardiothoracic surgery team devised a plan for the excision of the mass. Preoperative diagnostic cardiac catheterization revealed clear coronaries. The cardiothoracic surgery team successfully resected a 14 cm long tan-white polypoid fibrous mass, which was attached to the IVC near the atrial junction (Figure 2).

**Figure 3:**
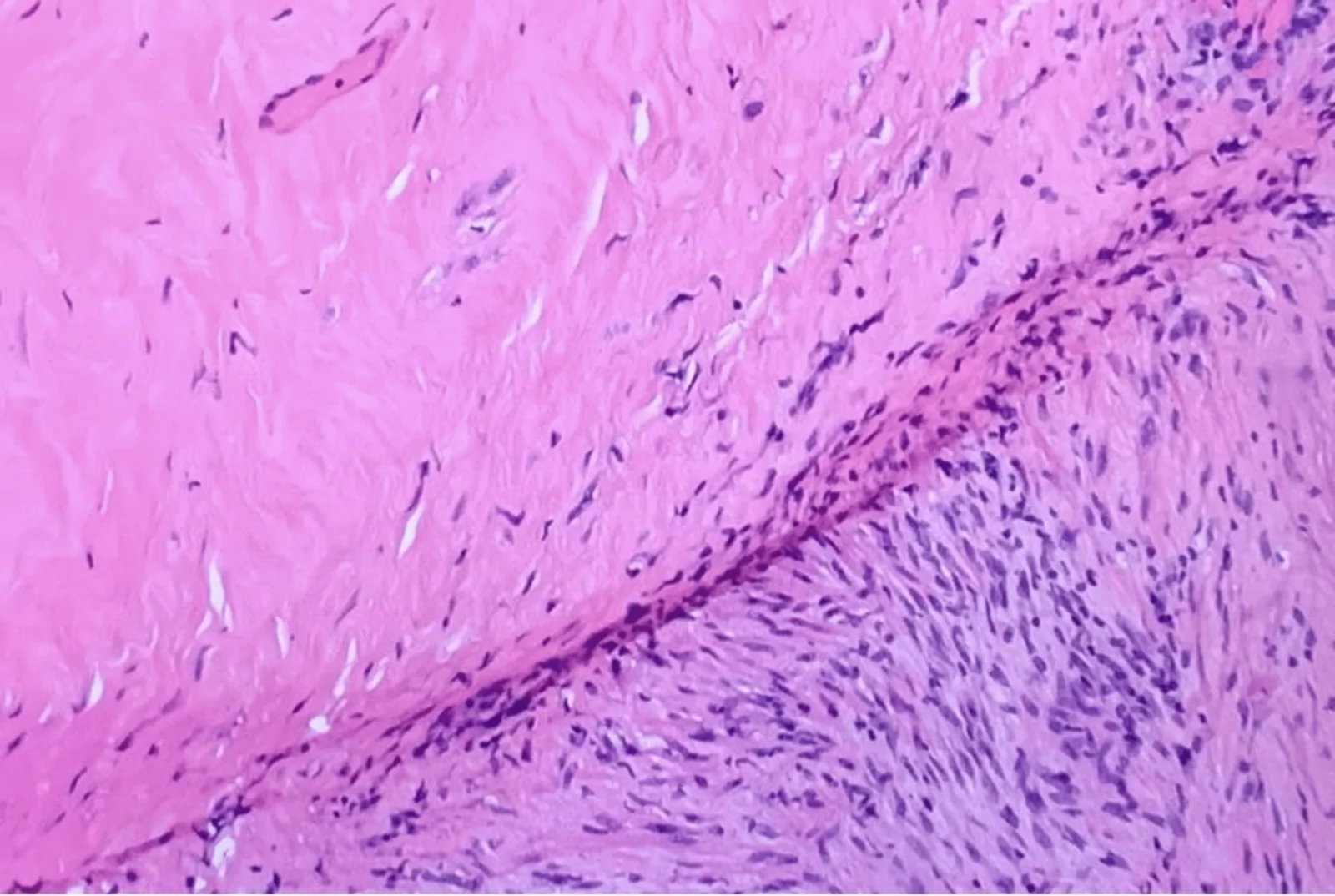
Hematoxylin and eosin stain demonstrating a hyalinized lesion with peripheral cellular proliferation adjacent to dense eosinophilic stromal material. No infiltrative growth, significant cytologic atypia, endothelial multilayering, brisk mitotic activity, or necrosis is identified.

**Figure 4:**
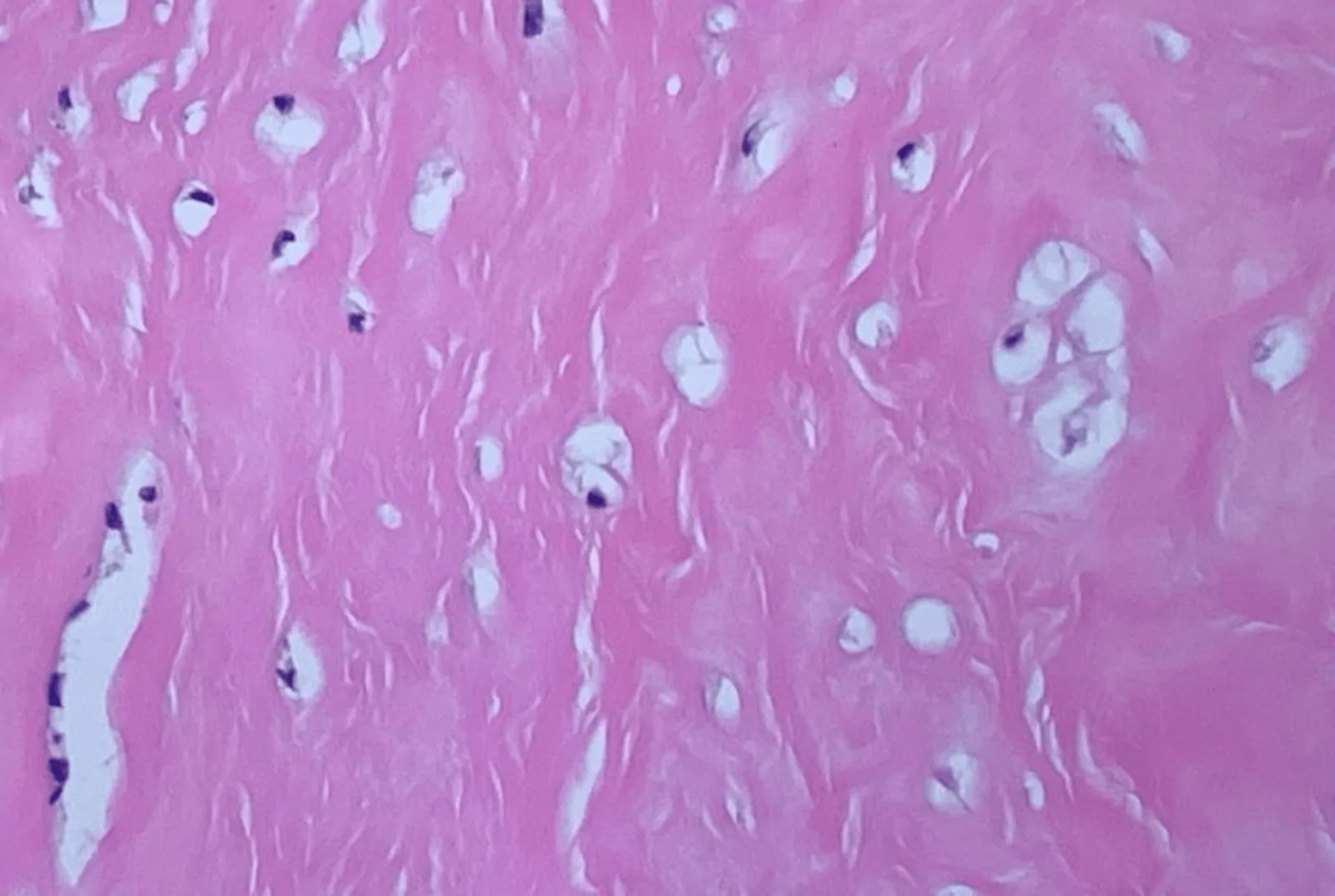
Higher-power hematoxylin and eosin stain showing marked hyalinization with vascular/lacunar spaces containing residual endothelial-lined channels.

**Figure 5:**
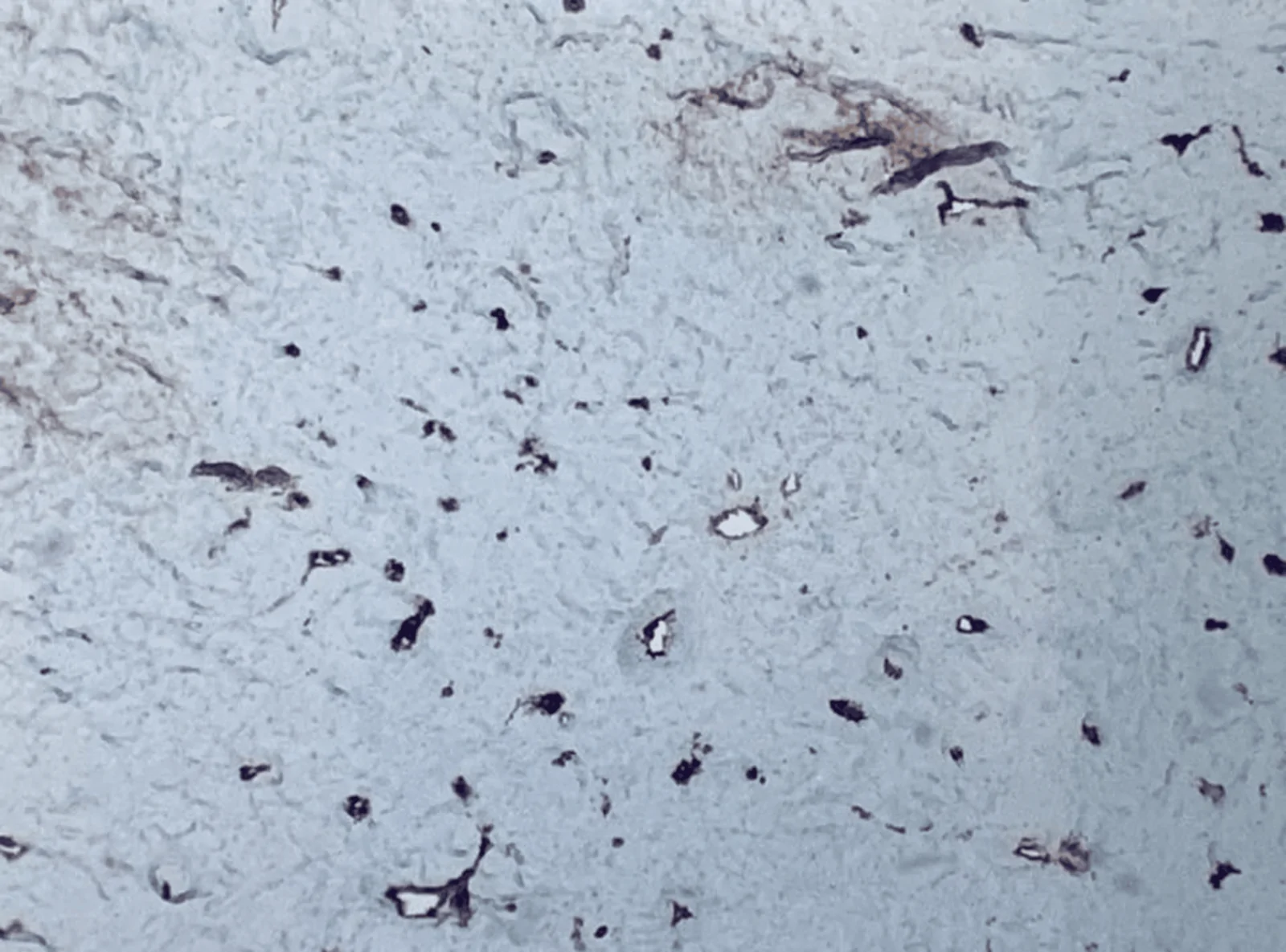
Higher-power view of CD31 immunohistochemical staining highlighting endothelial proliferation and vascular differentiation.

Immunohistochemical analysis demonstrated positivity for CD31 and smooth muscle actin (SMA), supporting endothelial and perivascular differentiation. The lesion was negative for calretinin and S100, excluding mesothelial and neural-derived neoplasms, respectively. These findings, in conjunction with characteristic histomorphologic features, supported the diagnosis of Masson’s tumor (IPEH) (Figures 3-5).

The patient was discharged home after an uneventful postoperative course. The patient was monitored on an outpatient basis for two years, with no evidence of cardiac mass recurrence during this period.

## Discussion

To our knowledge, our case represents the first reported case of an inferior vena cava Masson’s tumor to date. We conducted a comprehensive search on PubMed from 1976 to 2026 using the terms “Masson’s tumor,” “intravascular papillary endothelial hyperplasia,” “heart,” and “IVC.” Long et al. documented the first case of right ventricular Masson’s tumor complicated with saddle pulmonary embolism, which was also managed surgically [3]. Mantha et al. reported a case of left atrial Masson’s tumor leading to ischemic stroke, underscoring the necessity for anticoagulation therapy in such patients [2]. Park et al. in 1991 reported the first case of superior vena caval Masson’s tumor, which led to superior vena cava obstruction syndrome, which was thought to be lung carcinoma at admission of the patient [4]. In this case, histopathologic examination distinguished Masson’s tumor from angiosarcoma, its principal differential diagnosis. After complete resection, there was no recurrence of the tumor after two years of close follow-up.

## Conclusions

IPEH (Masson’s tumor) is a rare benign vascular lesion that can rarely present as an intracardiac mass and mimic more common cardiac tumors or thrombi. This case highlights the diagnostic challenges associated with right-sided cardiac masses, particularly when initial imaging studies provide conflicting anatomical impressions. Multimodality imaging, including TEE, was essential in accurately identifying the lesion’s origin from the IVC. Surgical excision remains both diagnostic and curative, with histopathological examination required for definitive diagnosis. Clinicians should consider Masson’s tumor in the differential diagnosis of intracardiac masses to avoid misdiagnosis as a malignant vascular neoplasm and to facilitate timely surgical management.
